# Parvovirus B19 Infection Presenting as Acute Tubulointerstitial Nephritis in an Immunocompetent Patient: A Case Report

**DOI:** 10.7759/cureus.93576

**Published:** 2025-09-30

**Authors:** Selim Benhadda, Yassir Tahri, Loubna Benamar, Naima Ouzeddoun, Tarik Bouattar

**Affiliations:** 1 Nephrology, Centre Hospitalo-Universitaire Ibn Sina, Rabat, MAR

**Keywords:** acute kidney injury, human parvovirus b19, infectious disease diagnosis, tubulointerstitial nephritis, viral nephropathy

## Abstract

A 21-year-old female patient with a history of *Helicobacter pylori-*associated gastritis and irregular menstrual cycles was admitted for fever, abdominal pain, vomiting, and stage 3 acute kidney injury (AKI). Three months earlier, she had presented with a pruritic rash and abdominal pain treated with amoxicillin-clavulanate, followed by recurrence of symptoms and deterioration. On admission, laboratory tests showed anemia, leukocytosis, thrombocytosis, elevated inflammatory markers, and mild proteinuria. An extensive infectious and autoimmune work-up was negative, except for positive IgM and IgG antibodies to parvovirus B19 (PVB19) and detectable viral DNA. Renal biopsy revealed acute tubulointerstitial nephritis without glomerular or vascular involvement. She was managed with supportive therapy, including hydration, antibiotics, and transfusion. Her renal function and clinical status progressively improved, with normalization of creatinine and inflammatory markers within three months. Although rare, PVB19 infection can present as acute interstitial nephritis even in immunocompetent adults. Recognition of this atypical presentation is crucial to avoid unnecessary immunosuppression and to anticipate spontaneous recovery.

## Introduction

Parvovirus B19 (PVB19) is a human pathogen belonging to the *Parvoviridae* family, typically responsible for erythema infectiosum (fifth disease) in children. In adults, it can cause more severe manifestations, including arthropathies in individuals with normal immune function, hydrops fetalis in pregnant women, transient aplastic crises in those with chronic hemolytic anemias, and chronic aplastic anemia in immunocompromised individuals [1].

Beyond hematological involvement, several reports have highlighted its association with acute kidney injury (AKI), often mediated by immune complex deposition, endothelial involvement, or tubular epithelial injury. These mechanisms explain the diverse renal presentations described, from postinfectious glomerulonephritis to vasculitis [2,3].

However, to our knowledge, tubular involvement has never been described except in a patient with hereditary spherocytosis [4]. Here, we present the case of an immunocompetent patient with a primary PVB19 infection, complicated by acute tubulointerstitial nephritis.

## Case presentation

A 21-year-old female patient presented with intense abdominal pain and vomiting. She had a history of *Helicobacter pylori*-associated gastritis and irregular menstrual cycles. Pregnancy was excluded at presentation by a negative test. Her medication history included occasional use of corticosteroids. She had no known allergies or consanguinity, and there was no family history of renal, hematologic, or autoimmune disorders.

Three months prior, she had reported a diffuse pruritic, erythematous rash involving the trunk and upper limbs, vomiting, and intense peri-umbilical abdominal pain, for which she received amoxicillin-clavulanate for seven days for suspected bacterial infection. The symptoms partially improved, but a month later, they recurred with fever and general deterioration. Laboratory tests in the emergency department revealed AKI with leukocytosis and markedly elevated inflammatory markers (C-reactive protein (CRP) 111 mg/L, procalcitonin 81 ng/mL), leading to her admission to the nephrology department (Table 1).

**Table 1:** Laboratory data summary UPCR: urine protein-to-creatinine ratio; WBC: white blood cells; RBC: red blood cells; MCV: mean corpuscular volume; MCHC: mean corpuscular hemoglobin concentration; LDH: lactate dehydrogenase; CRP: C-reactive protein; ANA: antinuclear antibodies; ANCA: anti-neutrophil cytoplasmic antibodies; GBM: glomerular basement membrane; PCR: polymerase chain reaction; HIV: human immunodeficiency virus; HBs: hepatitis B surface antigen; HBc: hepatitis B core antibody; HCV: hepatitis C virus; CMV: cytomegalovirus; IM: infectious mononucleosis

Parameters	Patient values at admission	Patient values at one month	Patient values at three months	Unit	Reference range
Serum creatinine	404	158.4	79.2	µmol/L	45–90
Serum albumin	32	36	40	g/L	35–50
Serum protein	65	72	78	g/L	60–83
UPCR	0.7	0.28	-	g/g	<0.2
Urine WBC	90	8	-	/mm³	<10
Urine RBC	5	2	-	/mm³	<10
Hemoglobin	6.2	10.9	12.5	g/dL	12.0–16.0
MCV	85	86	87	fL	80–100
MCHC	33	36	37	g/dL	32-36
Leukocytes	10740	5740	5500	10^9/L	4.5-11
Platelets	573	236	250	10^9/L	150–450
Haptoglobin	Undetectable	-	-	g/L	0.14–2.58
LDH	135	-	-	U/L	140–280
CRP	111	16.6	4	mg/L	<5
Procalcitonin	81	0.36	-	ng/mL	<0.05
Ferritin	146	205	-	ng/mL	22–300
C3	1.12	-	-	g/L	0.82–1.85
C4	0.33	-	-	g/L	0.15–0.53
Anticardiolipin antibodies	Negative	-	-	-	Negative
Anti-beta-2-glycoprotein-I antibodies	Negative	-	-	-	Negative
Lupus anticoagulants	Negative	-	-	-	Negative
ANA	Negative	-	-	-	Negative
Anti-DNA	Negative	-	-	-	Negative
ANCA	Negative	-	-	-	Negative
Anti-GBM	Negative	-	-	-	Negative
IgG anti-parvovirus B19	1.5	1.23	-	U/ml	<0.9
IgM anti-parvovirus B19	1.3	1.01	-	U/ml	<0.9
PCR parvovirus B19	4.42	3.18	0.05	log₁₀ copies/mL	<0.1
HIV 1/2 antigen/antibodies	Negative	-	-	-	Negative
HBs antigen (HBV)	Negative	-	-	-	Negative
Anti-HBs	Negative	-	-	-	Negative
Anti-HBc	Negative	-	-	-	Negative
Anti-HCV	Negative	-	-	-	Negative
IgG anti-CMV	107	-	-	UI/mL	<0.9
IgM anti-CMV	negative	-	-	UI/mL	<0.9
PCR CMV	1.924	1.2	-	log₁₀ copies/mL	<1.3
Genexpert	Negative	-	-	-	Negative
Syphilis serology	Negative	-	-	-	Negative
Rickettsioses serology	Negative	-	-	-	Negative
Syphilis serology	Negative	-	-	-	Negative
IM serology	Negative	-	-	-	Negative

On admission, the patient was hemodynamically stable and febrile (38.5°C). Physical examination revealed pale conjunctivae but no edema, cutaneous lesions, or arthritis. Abdominal, neurological, cardiovascular, and pulmonary examinations were unremarkable.

Laboratory investigations confirmed stage 3 AKI with a serum creatinine of 45.7 mg/L (404 μmol/L) and mild proteinuria. A hematological workup revealed normocytic, normochromic anemia, leukocytosis, and thrombocytosis. Inflammatory markers, including CRP and procalcitonin, were elevated. Extensive infectious screening revealed recent PVB19 infection (positive IgM and IgG) with positive viral polymerase chain reaction (PCR). Other viral and bacterial serologies were negative. Stool cultures and parasitological examinations were negative, excluding gastrointestinal infections.

**Figure 1:**
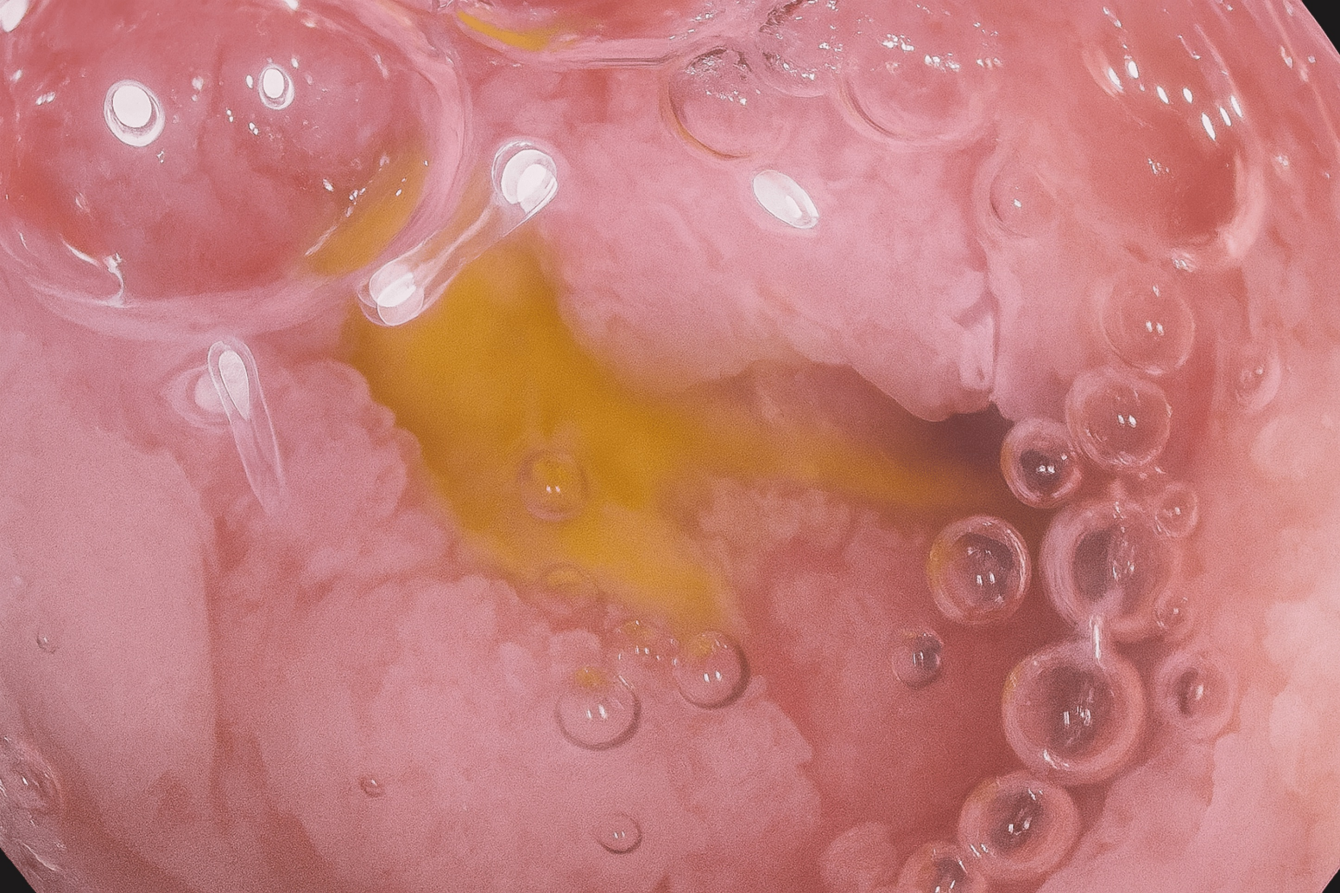
Fibroscopic image of a gastric ulcer with a fibrinous base

Further imaging studies included a thoraco-abdominal-pelvic CT scan, which was unremarkable, ruling out other sources of infection or malignancy (Video 1). Endoscopic evaluations included an esophagogastroduodenoscopy, which revealed a fundal ulcer likely due to corticosteroid use (Figure 1).

**Video 1:** Thoraco-abdomino-pelvic CT-scan of our patient

**Figure 2:**
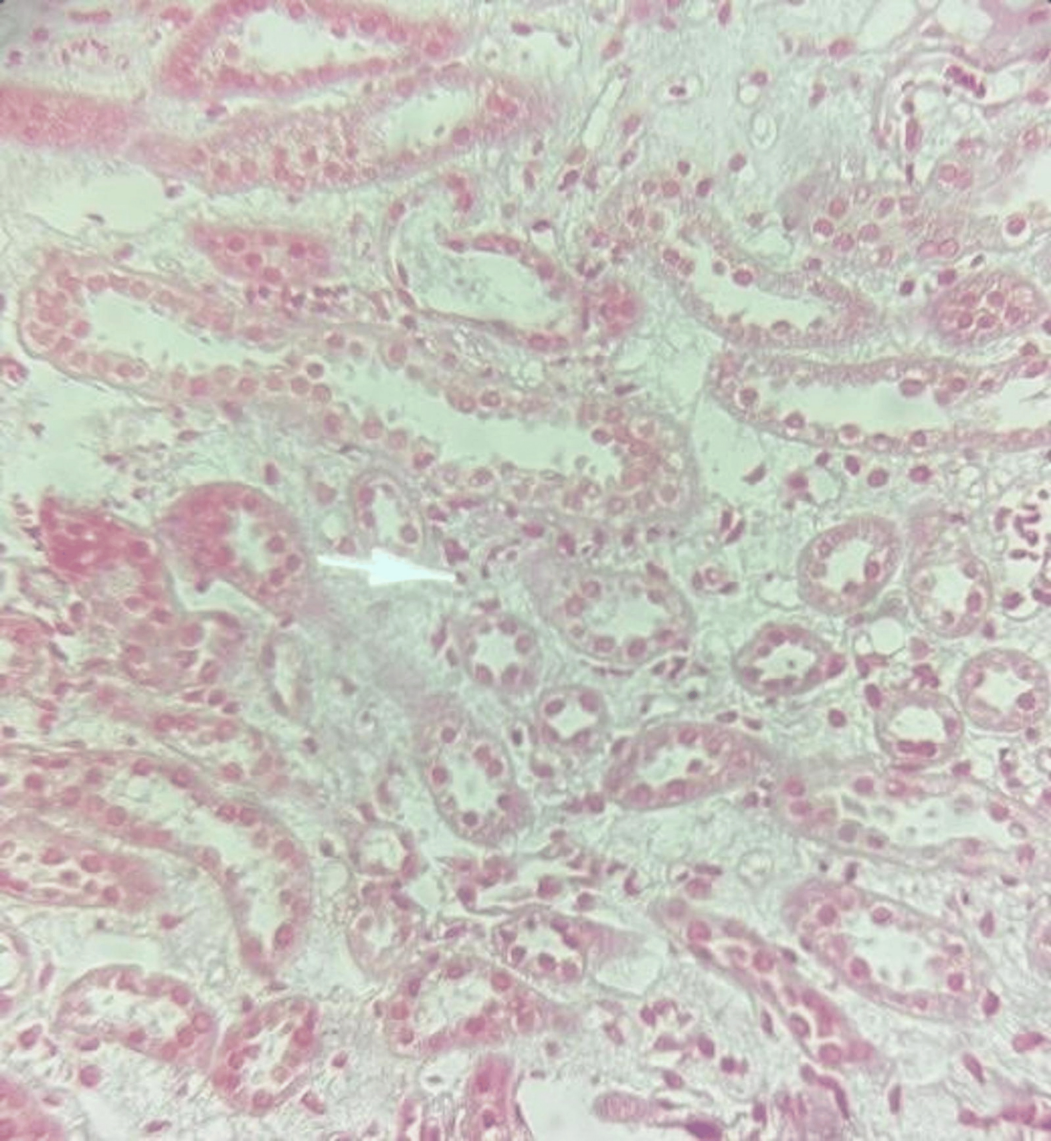
Light microscopy (H&E stain, ×400) Medium power field illustrating patchy interstitial inflammatory infiltrates and prominent edema. Tubular epithelial cells show signs of injury with cytoplasmic vacuolization and nuclear crowding (arrow). These findings are consistent with an active tubulointerstitial nephritis.

**Figure 3:**
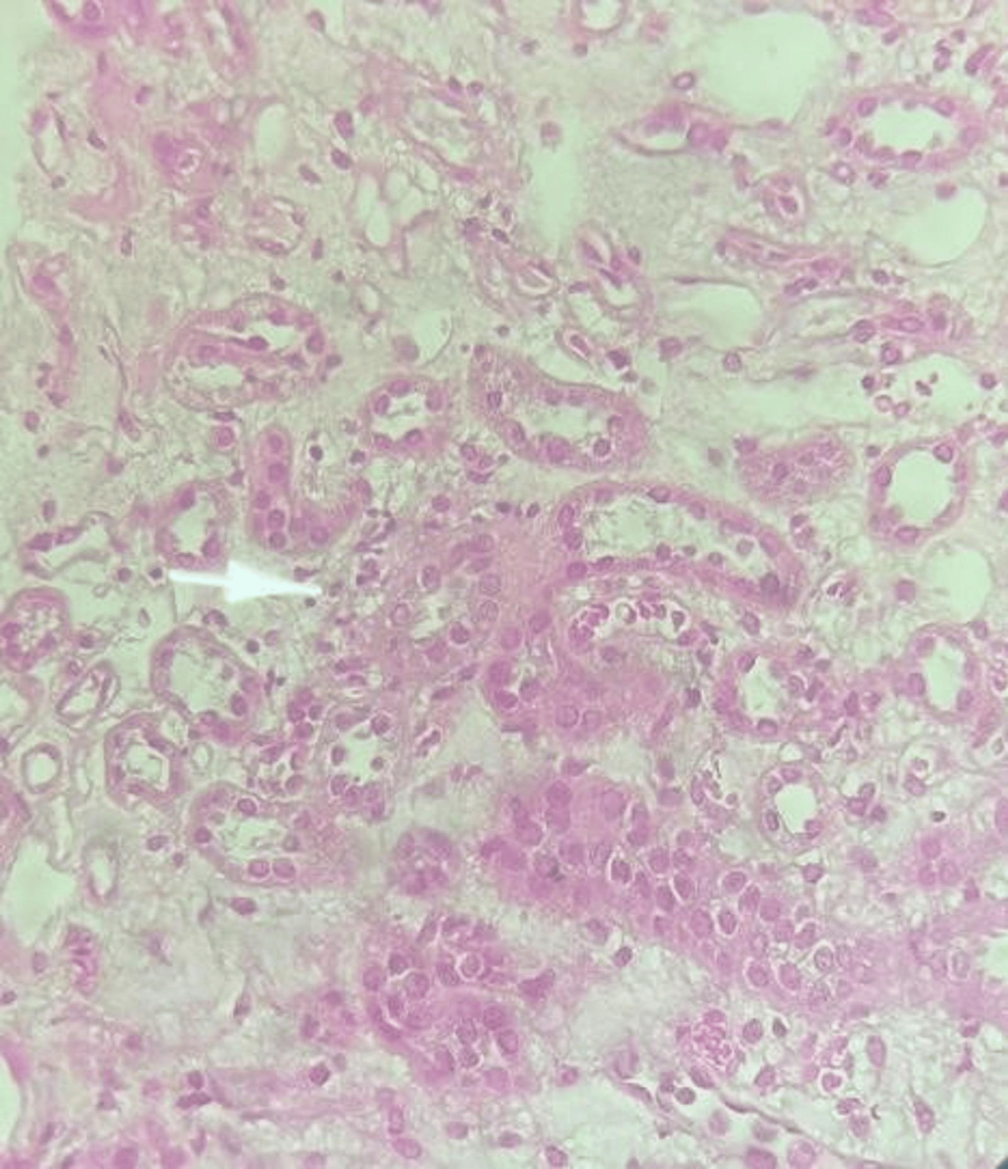
Light microscopy (H&E stain, ×400) High magnification image showing marked tubular epithelial cell injury with cytoplasmic vacuolization, loss of brush border, and nuclear crowding. There is evident interstitial edema and mononuclear inflammatory cell infiltration (arrow). These features are consistent with acute tubulointerstitial nephritis and highlight the severity of tubular damage.

A renal biopsy was performed. The cortex contained 12 glomeruli and showed acute tubulointerstitial nephritis. No vascular involvement, fibrosis, or immune deposits were noted (Figures 2, 3).

The patient was diagnosed with PVB19 infection complicated by acute tubulointerstitial nephritis. Management included hydration and acid-base correction. Empiric broad-spectrum antibiotics (ceftriaxone, levofloxacin, and metronidazole) were administered for 12 days, initiated in the setting of persistent fever and markedly elevated inflammatory markers, pending exclusion of bacterial infection. Supportive therapy included proton pump inhibitors, antiemetics, and transfusions for anemia.

The patient’s condition improved with the resolution of fever, abdominal pain, and inflammatory markers. Renal function showed gradual improvement, and anemia was corrected. Her renal function and clinical status progressively improved. 

## Discussion

PVB19 was first identified in 1975 by Yvonne Cossart during hepatitis B screening and named after a blood sample labeled "B19" [5]. Since its discovery, PVB19 has been associated with various renal manifestations, with acute postinfectious glomerulonephritis being the most commonly reported in immunocompetent individuals [3]. Additionally, cases of renal vasculitis, ranging from Henoch-Schönlein purpura to granulomatous polyangiitis, have also been documented [6].

The exact mechanism underlying the pathogenesis of PVB19-associated kidney disease remains unclear. Recent studies have revealed that PVB19's pathogenic effects are not limited to the hematopoietic system. Although the virus mainly infects erythroid progenitor cells in the bone marrow, leading to disorders like transient aplastic crisis and pure red cell aplasia, it can also invade endothelial, mesangial, and synovial cells [7]. This wider cellular tropism has been associated with diverse clinical conditions, such as myocarditis, arthritis, vasculitis, and glomerulonephritis [8]. Additionally, PVB19 can trigger immune complex formation and deposition in tissues, leading to kidney damage [7]. All of this may explain why postinfectious glomerulonephritis and membranoproliferative glomerulonephritis are among the most common renal manifestations of this viral infection [2,9]. In relation to our case, which demonstrated acute interstitial nephritis in our patient, Besse et al. have shown that the capsid antigen was positive for podocytes, parietal epithelial cells, and also tubular epithelial cells [2]. In another study, nephrotropic parvovirus has been linked to chronic tubulointerstitial nephritis in rodents, though its affinity for tubular epithelial cells remains unclear [10].

PVB19 infection was suspected in our patient after excluding all other possible causes of AKI and sepsis. No clinical, biological, or radiological evidence of infection was found, except for positive PVB19 serologies. In addition to this, the normochromic normocytic non-regenerative anemia associated with viral-like symptoms and spontaneous remission strongly suggests a PVB19 infection. We couldn’t provide any histological proof of direct renal injury induced by PVB19. The presence of PVB19 DNA in renal tissue detected by PCR is actually the gold standard of PVB19-associated nephropathy, but does not always confirm an active infection. The virus can persist in tissues for long periods without causing ongoing disease [11]. It is therefore essential to correlate PCR findings with clinical, histological, and serological data to determine the significance of viral presence in renal pathology. In a retrospective cohort study of 100 renal biopsies, Kauffmann et al. identified four cases of nephropathy associated with PVB19. All patients showed evidence of acute PVB19 infection, with both IgM and IgG antibodies present. Although tissue PCR used as the reference diagnostic method was positive in only one case, the temporal association between infection and kidney injury, along with the favorable spontaneous renal recovery in three patients (excluding one with chronic PVB19 infection), supported a causal relationship [3].

Enzyme-linked immunosorbent assay (ELISA) is the most widely used method for detecting virus-specific antibodies against capsid antigens in immunocompetent individuals [11]. In contrast, PCR is the diagnostic method of choice for immunocompromised patients, as it allows direct identification of viral DNA [11]. The specificity and sensitivity of PVB19-specific IgM assays are generally high, ranging between 70 and 100%, making them reliable for diagnosing acute infection [12]. However, false positives can occur, particularly in individuals with other autoimmune antibodies like rheumatoid factor and antinuclear antibodies [13]. Our patient had no positive antibodies, ruling out the possibility of a false positive.

Regarding therapeutic strategy, our patient experienced a spontaneous, favorable renal outcome four weeks after diagnosis. Indeed, most uncomplicated adult cases resolve spontaneously within two to four weeks. In the absence of a standardized treatment protocol, therapeutic strategies vary depending on the nature and severity of renal involvement. However, intravenous immunoglobulin (IVIg) therapy remains the cornerstone for managing persistent or severe PVB19 infection. Wolfromm et al. recommend initiating treatment when organ injury is not controlled [14]. In the series reported by Kauffmann et al., no immunosuppressive or IVIg therapy was administered to an immunocompetent patient with PVB19-induced lupus-like glomerulonephritis, and the patient achieved a spontaneous favorable renal outcome four weeks after diagnosis [3]. In our case, the follow-up strategy included serial monitoring of creatinine, hemoglobin, leukocyte count, CRP, and procalcitonin at one and three months, all showing progressive normalization. These findings are in line with our case, in which the patient also showed spontaneous improvement three months after diagnosis.

## Conclusions

PVB19 infection, though uncommon, should be recognized as a potential cause of acute tubulointerstitial nephritis in immunocompetent hosts. Its nonspecific manifestations frequently lead to a challenging diagnostic process. Early identification based on serological and molecular testing is essential to avoid unnecessary interventions. While most cases evolve favorably with supportive care alone, severe or refractory presentations may warrant immunomodulatory therapy, particularly IVIg, adapted to the patient’s clinical and histological profile. By documenting such a rare manifestation in an immunocompetent adult, this case contributes to broadening awareness of PVB19 as a potential cause of acute interstitial nephritis.
